# The History, Diagnosis and Treatment of the Fevers of the United States

**Published:** 1857-09

**Authors:** 


ART. IX.—*The History, Diagnosis and Treatment of the Fevers of the United States.* By ELISHA BARTLETT, M. D., late Professor of Materia Medica and Medical Jurisprudence in the College of Physicians and Surgeons of the State of New York, etc., etc. Fourth edition, Revised. By A. CLARK, M. D., Professor of Pathology and Practical Medicine in the College of Physicians and Surgeons of the State of New York. Philadelphia: BLANCHARD & LEA. 1857. 8vo. pp. 610.

It would be more than an act of supererogation, at this day, to invite the attention of our readers to the merits of the work on fever by the late Dr. Bartlett. It would be a reflection on their information and intelligence. The work has become classical in medical literature. The value placed upon it by the profession is shown by the demand for a fourth edition, the first having been issued ten years ago.

The present edition has been prepared since the death of the lamented author. The duty was confided by the author himself during his last illness, to his intimate friend, Prof. Clark; and to no one could it have been more appropriately committed. To edit the work was with Prof. Clark a labor of love. Moreover, his own studies eminently fitted him to fulfil the trust,—on the one hand to his endeared friend, and, on the other hand, to the medical profession.

Five years only have elapsed since the publication of the third edition of this work; but in that time, to quote the language of the editor, "new facts have been observed, and new opinions have been expressed, which both add to our knowledge and suggest new topics for investigation." Hence, although' as the editor states, scarcely a dozen phrases have been changed, considerable matter has been added in the present edition.

Believing, as we do, that the facts and doctrines contained in this work are not likely soon to undergo essential alterations, as medical knowledge advances, we predict that it will long remain, as it now is, preëminently the standard work on the fevers of the United States.

For sale by PHINNEY & Co. *

